# The Error of Estimated GFR in Type 2 Diabetes Mellitus

**DOI:** 10.3390/jcm8101543

**Published:** 2019-09-26

**Authors:** Sergio Luis-Lima, Tomás Higueras Linares, Laura Henríquez-Gómez, Raquel Alonso-Pescoso, Angeles Jimenez, Asunción María López-Hijazo, Natalia Negrín-Mena, Candelaria Martín, Macarena Sánchez-Gallego, Sara Judith Galindo-Hernández, Raquel Socas Fernández del Castillo, Manuel Castilla-Marrero, Santiago Domínguez-Coello, Vanesa Vilchez de León, Rafael Valcárcel-Lopez, Nerea Insausti-Garmendia, Beatriz Escamilla, Sara Estupiñán, Patricia Delgado-Mallén, Ana-María Armas-Padrón, Domingo Marrero-Miranda, Ana González-Rinne, Rosa María Miquel Rodríguez, María Angeles Cobo-Caso, Laura Díaz-Martín, Federico González-Rinne, Alejandra González-Delgado, Marina López-Martínez, Alejandro Jiménez-Sosa, Armando Torres, Esteban Porrini

**Affiliations:** 1 Department of Nephrology and Hypertension, IIS-Fundacion Jimenez Diaz, 28040 Madrid, Spain; luis.lima.sergio@gmail.com; 2 Centro de Salud Tejina, 38260 La Laguna, Tenerife, Spain; tomashigueras59@gmail.com (T.H.L.); mccastilla59@gmail.com (M.C.-M.); 3 Endocrinology Department, Hospital Universitario de Canarias, 38320 La Laguna, Tenerife, Spain; lunavera79@hotmail.com (L.H.-G.); rapescoso@hotmail.com (R.A.-P.); msga88@gmail.com (M.S.-G.); sagahe212@gmail.com (S.J.G.-H.); 4 Centro de Salud de Puerto de la Cruz- Casco Botánico, 38400 Puerto de la Cruz, Tenerife, Spain; angelesjimenez2006@gmail.com (A.J.); candymcp@gmail.com (C.M.); 5 Centro de Salud de La Vera, 38400 Puerto de la Cruz, Tenerife, Spain; asunlopez9@hotmail.com; 6 Research Unit Department, Hospital Universitario de Canarias, 38320 La Laguna, Tenerife, Spain; natalianegrinmena@gmail.com (N.N.-M.); lauradiazmart@gmail.com (L.D.-M.); f.gonzalez.rinne@gmail.com (F.G.-R.); ajimenezsosa@gmail.com (A.J.-S.); 7 Centro de Salud de Las Dehesas, 38300 La Orotava, Tenerife, Spain; caquela72@hotmail.com (R.S.F.d.C.); girlvane@hotmail.com (V.V.d.L.); 8 Centro de Salud de La Victoria de Acentejo, 38380, La Victoria de Acentejo, Tenerife, Spain; sdominguezc@telefonica.net; 9 Centro de Salud de Santa Ursula, 38390 Santa Ursula, Tenerife, Spain; rafaelvl.mfyclalaguna@gmail.com; 10 Centro de Salud La Cuesta, 38320 La Laguna, Tenerife, Spain; nereainsa@gmail.com (N.I.-G.); anaarmaspadron@ecihucan.es (A.-M.A.-P.); 11 Nephrology Department, Hospital Universitario de Canarias, 38320 La Laguna, Tenerife, Spain; bescab@gmail.com (B.E.); saraesto@hotmail.com (S.E.); patridelma@gmail.com (P.D.-M.); dmarrero72@hotmail.com (D.M.-M.); rinneanag@yahoo.es (A.G.-R.); romiquelro@gmail.com (R.M.M.R.); mcobcas@gmail.com (M.A.C.-C.); 12 Central Laboratory, Hospital Universitario de Canarias, 38320 La Laguna, Tenerife, Spain; alejandra.gd88@gmail.com; 13 Nephrology Department, Hospital Germans Trias i Pujol, 08916 Badalona, Spain; marina.lmga@gmail.com; 14 Internal Medicine Department, ITB: Instituto de Tecnologías Biomédicas, Universidad de La Laguna, 38320 La Laguna, Tenerife, Spain; atorresram@gmail.com

**Keywords:** Type II diabetes mellitus, renal disease, estimated glomerular filtration rate, measured glomerular filtration rate, plasma clearance of iohexol

## Abstract

Type 2 diabetes mellitus represents 30–50% of the cases of end stage renal disease worldwide. Thus, a correct evaluation of renal function in patients with diabetes is crucial to prevent or ameliorate diabetes-associated kidney disease. The reliability of formulas to estimate renal function is still unclear, in particular, those new equations based on cystatin-C or the combination of creatinine and cystatin-C. We aimed to assess the error of the available formulas to estimate glomerular filtration rate in diabetic patients. We evaluated the error of creatinine and/or cystatin-C based formulas in reflecting real renal function over a wide range of glomerular filtration rate (from advanced chronic kidney disease to hyperfiltration). The error of estimated glomerular filtration rate by any equation was common and wide averaging 30% of real renal function, and larger in patients with measured glomerular filtration rate below 60 mL/min. This led to chronic kidney disease stages misclassification in about 30% of the individuals and failed to detect 25% of the cases with hyperfiltration. Cystatin-C based formulas did not outperform creatinine based equations, and the reliability of more modern algorithms proved to be as poor as older equations. Formulas failed in reflecting renal function in type 2 diabetes mellitus. Caution is needed with the use of these formulas in patients with diabetes, a population at high risk for kidney disease. Whenever possible, the use of a gold standard method to measure renal function is recommended.

## 1. Introduction

Type 2 diabetes is a major health problem affecting 8.5% of the world population http://apps.who.int/iris/bitstream/10665/204871/1/9789241565257_eng.pdf?ua=1 (accessed 12 June 2019), and consuming about 1.8% of the global domestic product [1]. In the US and in Europe, type 2 diabetes accounts for 30% to 50% of the cases of end stage renal disease https://www.renalreg.org/reports/2017-twentieth-annual-report (accessed 12 June 2019) [2]. Importantly, this prevalence did not change in the last 20 years, despite new therapies and intensive care of diabetic patients [3]. This makes the study of diabetic-related renal disease an important issue in clinical medicine and nephrology.

In clinical practice, renal function is evaluated by formulas that provide estimations of glomerular filtration rate (GFR). These formulas are mathematical algorithms based on endogenous markers like serum creatinine or cystatin-C, and other variables such as weight, height, and gender. More than 60 equations have been developed in the last 60 years. However, the capacity of formulas to reflect real renal function is a matter of debate. Different studies in type 2 diabetes have evaluated the agreement between estimated GFR and measured GFR showing contradictory results [4,5,6,7,8,9,10,11,12,13,14]. Some publications support the use of formulas [4,6,8], whereas other studies reported an unacceptable wide error [5,7,11,12,13,15]. However, not all the formulas have been tested, in particular, the most recent cystatin-C-based equations. Whether these new algorithms outperform the traditional formulas based on the creatinine in type 2 diabetes is unclear [8,10,11]. Thus, the utility of eGFR in type 2 diabetes is still unclear, in particular, those new equations based on cystatin-C or the combination of creatinine and cystatin-C. Whether formulas are reliable or not is a relevant issue, since errors in the estimation of GFR may limit the evaluation of renal function in single patients or jeopardize the evaluation of positive effects of new therapies aimed at preventing renal function loss in this population.

The present work aimed to study the agreement of a broad group of creatinine and/or cystatin-C based formulas with real renal function in a large population of diabetic patients over a wide range of GFR (from advanced chronic kidney disease (CKD) to hyperfiltration).

## 2. Methods

### 2.1. Patients

We performed a cross-sectional study of 475 adults (>18 years) with type 2 diabetes attending the outpatient clinics of the Nephrology and Endocrinology departments at the Hospital Universitario de Canarias (HUC), in whom we performed the plasma clearance of iohexol from 2012 to February 2018. All patients signed an informed consent form, and the study was approved by the Ethics Committee of the HUC.

### 2.2. Measured GFR

GFR was determined by the plasma clearance of iohexol as shown previously [16,17]. In brief, on the morning of the study, 5 mL of iohexol (Omnipaque 300, GE-Healthcare, Chicago, IL, USA) were injected intravenously for 2 min. Afterwards, venous or capillary blood was obtained by finger prick at 2, 3, 4, 5, 6, 7, and 8 h for patients with eGFR ≤40 mL/min; or at 2, 2.5, 3, 3.5, and 4 h for those with eGFR >40 mL/min. The concentrations of iohexol were determined in plasma or dried blood spot (DBS) testing [16,17]. DBS testing can be considered interchangeable with plasma analysis [17].

### 2.3. Estimated GFR by Formulas

Simultaneously to the plasma clearance of iohexol, serum creatinine and cystatin C were determined to calculate eGFR. All the studied formulas are shown in: www.ecihucan.es/lfr/apps/documents/egfr_formulas_v2019feb.pdf (accessed 12 June 2019).

The agreement between formulas and measured GFR was evaluated with the formulas adjusted and unadjusted for body surface area (BSA). When estimated GFR was already adjusted we reversed the adjustment of the result by applying the following formula (GFR adjusted = GFR unadjusted/BSA × 1.73). On the other hand, when estimated GFR was unadjusted, we adjusted the result by applying the formula (GFR unadjusted = GFR adjusted × BSA/1.73). BSA was calculated by Du-Bois and Du-Bois formula [18].

### 2.4. Biochemistry

Creatinine (mg/dL) was measured by enzymatic assay (IDMS-traceable creatinine) using the cobas c711 module (Roche Diagnostics). Cystatin C (mg/L) was measured by immunonephelometry (BN II System-Siemens Healthcare Diagnostics) calibrated with ERM-DA471/IFCC.

### 2.5. Statistical Analysis

#### 2.5.1. Agreement between eGFR and mGFR

The agreement between estimated and measured GFR was assessed by specific statistics for continuous data, including the concordance correlation coefficient (CCC), total deviation index (TDI), coverage probability (CP) [19,20,21], and Bland-Altman limits of agreement (LA) [22]. The CCC varies from 0 to 1 and combines meaningful components of accuracy and precision. A CCC >0.90 reflects optimal concordance between measurements. The TDI captures a large proportion of data within a boundary for allowed differences between 2 measurements. Empirical TDI was calculated for a theoretical TDI of 10% and a CP of 90%. We defined a priori that acceptable bias between eGFR and mGFR should be at least 10%, and that 90% of the estimations should be included within these limits. This is based on previous reports and the reproducibility of measured GFR considering different methods [15]. Coverage probability varies from 0 to 1 and estimates whether a given TDI is less than a prespecified fixed percentage. Bland-Altman plots show the relationship between the difference between target and observed measurements and the mean of both [22]. The smaller the limits of agreements are, the higher the degree of agreement between measurements.

#### 2.5.2. Error in the Classification of CKD Stages

The patients were classified in CKD stages according to the K/DOQI guidelines [23]. The agreement between the classification in CKD stages based on mGFR or eGFR (for a representative number of 11 formulas) were analyzed. *True Positive Cases* represented the individuals correctly classified by the estimated GFR in the CKD stage defined by measured GFR; *False Positive Cases* represented the subjects incorrectly classified by a formula in a given stage defined by measured GFR; and (c) *Missing Cases* represented the patients that were misclassified in a higher or lower stage of CKD. True positive and false positive cases represented 100% of the cases defined by a formula in a stage of CKD, and true positive and missing cases represented 100% of the cases defined by measured GFR in a stage of CKD.

#### 2.5.3. Accuracy of Estimated GFR with Measured GFR

Finally, for comparability with other studies, we assessed the accuracy of each formula as the proportion of eGFR results within 10% (P10) and 30% (P30) of mGFR.

For agreement analyses, we designed and implemented a software (AGP Agreement Program v1.0 IGEKO, SP) available at: www.ecihucan.es/lfr/apps/?dir=agreement_installer (accessed 12 June 2019).

In addition, we used SPSS Statistics for Windows, version 17.0 (SPSS Inc., Chicago, IL, USA), and MedCalc Statistical Software version 13.0.2 (MedCalc Software bvba, Ostend, Belgium).

## 3. Results

### 3.1. Patients

About 70% of the patients were male, age averaged 63.3 ± 11.2 years (Table 1). Body mass index (BMI) averaged 29.7 ± 6.8 kg/m^2^. Median proteinuria was 426.1 (1511.7) mg/24 h.

Measured GFR unadjusted by BSA averaged 57.1 ± 36.3 mL/min (8.5 to 180.5 mL/min), whereas mGFR adjusted by BSA averaged 51 ± 31.3 mL/min/1.73 m^2^ (8.6 to 150.7 mL/min/1.73 m^2^) (Table 1). Based on measured GFR unadjusted by BSA, 86 subjects (18%) were classified in CKD-1; 94 (20%) in CKD-2; 156 (33%) in CKD-3; 116 (24%) in CKD-4 and 23 (5%) in CKD-5 (Table 1).

### 3.2. Agreement between Measured GFR and Estimated GFR

#### 3.2.1. Creatinine Based Formulas

TDI averaged 75%, ranging from 52% to 144% for the Lund-1 and Mogensen equations respectively (Table 2). For example, the aMDRD formula had a TDI of 58%, meaning that 90% of estimations erred from −58 to 58% of measured GFR. A similar TDI was observed for broadly used formulas like the CKD-EPI (58%), MCQ (77%) and Cockcroft-Gault (70%), or more recent ones like Lund-Malmö (Rv) (55%). CCC averaged 0.87, reflecting moderate precision and accuracy, ranging from 0.75 to 0.92, for the Mogensen and Lund-Malmö (Rv) equations, respectively (Table 2). Finally, CP averaged 20, indicating that more than 80% of the estimations had an error greater than ± 10% (Table 2). Bland and Altman’s plots showed wide limits of agreement, averaging from +30 to −45 mL/min, which indicates very poor agreement between estimated and measured GFR (Supplementary Figure S1).

#### 3.2.2. Cystatin-C Based Formulas

Cystatin-C was available in the 390 of 475 patients. TDI averaged 65%, ranging from 46% to 118% for the Stevens-2 and Perkins equations respectively (Table 2). For example, the Rule formula had a TDI of 60%, indicating that 90% of the estimations erred from −60% to 60% of measured GFR. A similar TDI was observed for other formulas like CKD-EPI cystatin-C (51%) or Rule cystatin-C (60%). CCC averaged 0.89, reflecting moderate precision and accuracy, ranging from 0.74 to 0.92, for Stevens-2 and CKD-EPI equations (Table 2). Finally, CP averaged 25, indicating that more than 75% of the estimations had an error greater than ± 10% of measured GFR (Table 2). Bland and Altman’s plots showed wide limits of agreement, averaging from +25 to −25 mL/min, which indicates poor agreement between eGFR and mGFR (Supplementary Figure S1)

#### 3.2.3. Creatinine and Cystatin-C Based Formulas

TDI averaged 55%, ranging from 43% to 78% for the Stevens and full age spectrum (FAS) equations respectively (Table 2). For example, the CKD-EPI formula had a TDI of 45%, and thus, 90% of the estimations erred from −45% to +45% of measured GFR. A similar TDI was observed for other equations like: Ma (54%), Stevens (44%), and FAS combi (78%). CCC averaged 0.91, reflecting a moderate level of precision and accuracy, ranging from 0.85 to 0.95, for the FAS and Stevens equations, respectively (Table 2). Finally, CP averaged 25%, indicating that more than 75% of the estimations had an error greater than ± 10% of measured GFR (Table 2). Bland and Altman’s plots showed wide limits of agreement, averaging from +20 to −30 mL/min, which indicates low agreement between estimated and measured GFR (Supplementary Figure S1)

Similar results were observed in the analysis of agreement between estimated and measured GFR using data adjusted by BSA (Supplementary Figure S1). Moreover, no major differences were observed in the agreement between estimated and measured GFR for a given formula, with or without BSA adjustment (Table 2 and Supplementary Table S1).

### 3.3. Subgroups of GFR

The error of formulas, either creatinine and/or cystatin-C based, was larger in patients with measured GFR <60 mL/min than in those with higher GFR (Supplementary Table S2). For example, the CKD-EPI creatinine-based formula showed a TDI of 70% in patients with measured GFR <60 mL/min, whereas the TDI was 48% in patients with GFR ranging from 60 to 90 mL/min and 24% for those individuals with measured GFR values >90 mL/min. Similar results were observed for the other formulas equations (Supplementary Table S2).

### 3.4. The Error in the CKD Classification

In general, one patient out of three was incorrectly classified either in lower or higher CKD stages. For example, for patients in CKD stage 3, false positive cases averaged 30%, true positive about 55%, and missing cases 45%. In this stage, the CKD-EPI creatinine-based formula incorrectly classified 27% of the patients (false positive cases); the CKD-EPI cystatin-C-based equation correctly classified 59% of the individuals (true positive) and the CKD-EPI creatinine and cystatin-C-based algorithm misclassified 47 of the subjects in a higher or lower stage of CKD (missing cases) (Supplementary Figure S2).

### 3.5. Hyperfiltering Patients

For unadjusted GFR values by BSA, hyperfiltration (GFR > 120 mL/min) was observed in 37 patients (8%). Equations failed to detect hyperfiltration in about 20% of the cases (Supplementary Table S1). For adjusted GFR values by BSA, hyperfiltration (GFR > 120 mL/min) was observed in 13 patients (2.7%). Formulas failed to detect hyperfiltration in about 60% of the subjects (Supplementary Table S3).

### 3.6. Examples of Under or Overestimation of GFR

Table 3 illustrates the error of estimated GFR through a wide range of measured GFR (8.5 to 180.6 mL/min). The cases were selected in pairs of similar measured GFR to show that the error of estimated GFR occurs at random.

Cases 1 and 2 had measured GFR of 17 mL/min and almost all the formulas underestimated (case 1) or overestimated real GFR (case 2). In addition, the same equations could either be overestimated or underestimated GFR for a similar measured GFR. For cases 9 and 10 (measured GFR of 100 mL/min), creatinine based formulas underestimated (case 9) and overestimated true GFR (case 10), whereas cystatin-C based equations showed opposite results. This determined an erroneous classification in a higher or lower CKD stage for cases 7 and 8 (mGFR = 67–68 mL/min; CKD stage 2), but all the formulas classified case 7 as CKD 3, whereas case 8 was defined as CKD 1 (GFR >90 mL/min) only for creatinine-based equations (Table 3).

**Figure 1:**
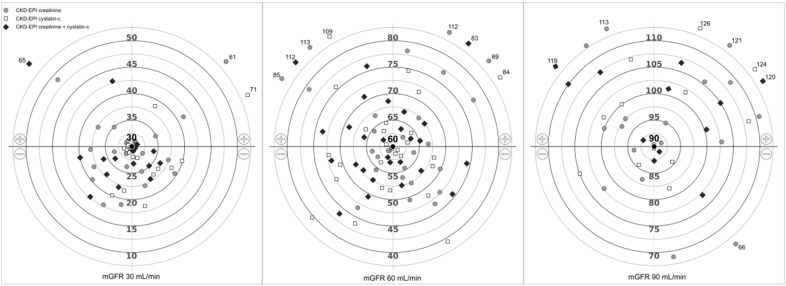
Bias of the three Chronic Kidney Disease Epidemiology Collaboration (CKD-EPI) equations (creatinine and/or cystatin-C based) for those patients with measured GFR of 30 mL/min (**left**), 60 mL/min (**middle**), and 90 mL/min (**right**).

Figure 1 represents the bias of the three CKD-EPI equations (creatinine and/or cystatin-C based) for those patients with measured GFR of 30 mL/min (Figure 1, left), 60 mL/min (Figure 1, middle), and 90 mL/min (Figure 1, right). Formulas tended to underestimate mGFR of 30 mL/min (Figure 1, left), either over and underestimated mGFR of 60 mL/min (Figure 1, middle), and mainly overestimated mGFR of 90 mL/min (Figure 1, right). Some estimations showed extreme variability: For a true GFR of 30 mL/min (Figure 1, left) estimated GFR could be 61 mL/min (CKD-EPI creatinine), 71 mL/min (CKD-EPI cystatin-C), or 65 mL/min (CKD-EPI creatinine and cystatin-C); for mGFR of 60 mL/min (Figure 1, middle), estimated GFR could be 39 (CKD-EPI cystatin-C) or about 110 mL/min for the three CKD-EPI algorithms; for measured GFR of 90 mL/min (right), eGFR could be 66 mL/min or about 120 mL/min for the CKD-EPI equations.

### 3.7. Accuracy of Estimated GFR with Measured GFR

The proportion of estimated GFR results within 10% (P10) of measured GFR values (mL/min) were 23.7%, 28.7%, and 31.3% for creatinine, cystatin-C and creatinine, and cystatin-C based formulas, respectively. These percentages were 61.2%, 69.7%, and 73.2% respectively, for the proportion of eGFR results within 30% of mGFR values (P30) (Supplementary Table S4). Similar results were observed for mGFR values adjusted by BSA (mL/min/1.73 m^2^) (Supplementary Table S5). An error larger than ±10% was particularly frequent in subjects with proven renal disease.

## 4. Discussion

We observed that the error of estimated GFR in type 2 diabetes by any equation based on creatinine and/or cystatin-C (1) was common and wide, (2) averaged 40% of real renal function, (3) was larger in patients with measured GFR below 60 mL/min compared to those above this level, and (4) lead to extreme variations between estimated and measured GFR. In addition, ~30% of the individuals were misclassified in CKD stages, and 25% of those with hyperfiltration were not diagnosed. Finally, the error was at random, since for a similar value of measured GFR in different patients, the same formula provided estimations that either over or underestimated measured GFR.

To evaluate the error of estimated GFR in type 2 diabetes, we analyzed a representative group of patients with a wide range of renal function, from 8.5 to 180.6 mL/min, normo, micro, and macroalbuminuria, with or without diabetic nephropathy, including renal transplanted patients. In addition, GFR was estimated with 61 formulas based on creatinine and/or cystatin-C, including old and more recent equations.

Our major finding was that estimated GFR calculated by any formula proved to be unreliable in type 2 diabetes. The error of the formulas was extremely large, with 90% of the estimations showing an error of about ± 30% of real GFR, as reflected both by large TDIs and wide limits of agreement (Table 2 and Supplementary Table S1). Moreover, in the remaining 10% of the cases, the error was even larger. For example, case 3 of Table 3 showed estimated GFR values of about 15 mL/min based on aMDRD, Mayo Clinic Quadratic (MCQ), and CKD-EPI equations, whereas measured GFR was 27 mL/min (45% of underestimation) (Table 3). Similarly, case 7 showed mGFR value of 67 mL/min, while CKD-EPI formulas based on creatinine and/or cystatin-C estimated GFR with values about 45 mL/min (33% of underestimation) (Table 3).

The clinical consequences of this error are relevant. Over and underestimation of measured GFR were particularly frequent (Table 3 and Figure 1), a fact that limits the evaluation of renal function. Moreover, the error of estimated GFR was at random, since in those subjects with similar measured GFR, the formulas gave opposite values of renal function. For example, cases 5 and 6 had a similar mGFR value of about 48 mL/min, whereas CKD-EPI formula overestimated GFR of 55 mL/min for case 5, and underestimated GFR of 28 mL/min for case 6. The misclassification of subjects in CKD stages was common, with one third of the patients incorrectly classified in lower or higher stages. For example, case 4 showed mGFR of 28 mL/min corresponding to CKD stage 4, whereas almost all the analyzed formulas classified this case as CKD stage 3 (Table 3). Conversely, case 6 had mGFR of 49 mL/min, defined as CKD stage 3, whereas some creatinine based formulas classified this case as CKD stage 4 (Table 3). Finally, hyperfiltration, an early marker of diabetic nephropathy, was undetected by formulas in about 20% of the cases (Supplementary Table S3).

The causes of the failure of formulas are not completely understood. Formulas are mathematical algorithms mainly based on endogenous (creatinine and cystatin-c), and other variables like age, weight, and gender. However, these markers have limitations in reflecting real GFR [15]. Tubular secretion of creatinine increases proportionally to the decrease in GFR, leading to a false overestimation of renal function [24]. In addition, creatinine can be metabolized or excreted via extra-renal routes, particularly in the gastrointestinal tract by gut flora, which may contribute to the overestimation of GFR [25]. Finally, muscle mass and protein intake may influence the levels of creatinine. Reduced muscle mass leads to lower production of creatinine and thus overestimation of GFR [26]. Increased muscular mass or muscle consumption may determine a rise in serum creatinine and, therefore, an underestimation of GFR. Dietary protein deficiency leads to reduced availability of the creatine precursors like arginine and glycine which may contribute overestimation of renal function [27].

High levels of cystatin-C are associated with age, male, gender, black race, weight, height, cigarette smoking, and subclinical inflammation, central adiposity, diabetes, and metabolic syndrome [28]. In fact, high cystatin-C predicts the appearance of diabetes. Thus, high levels of cystatin-C levels may indicate the clinical situations listed above and not necessarily reduced renal function.

Some equations are more used than others and deserve an especial consideration. The MDRD equation was developed in patients with CKD, thus, its performance is expected to be better in this population. However, this equation showed a comparable TDI with respect to other equations developed in a population with the whole spectrum of GFR like CKD-EPI formulas [12]. Similar results were reported in other studies [15], reflecting that the MDRD equation does not outperform other equations not designed in patients with GFR <60 mL/min. On the other hand, more modern equations were developed in populations covering a wide range of renal function, from advanced CKD (GFR < 15 mL/min) to normal GFR (GFR >90 mL/min) in subjects with or without renal disease [15]. Nevertheless, the agreement between these equations and mGFR proved to be as poor as older equations such as Effersoe or Cockcroft-Gault. In fact, the Effersöe formula from 1957, performed similarly (TDI = 61%) as the more recent equations such as CKD-EPI creatinine based (TDI = 58%) or full age spectrum (FAS) algorithms (TDI >75%) (Table 2). This lack of improvement was reported in other studies [15].

On the other hand, MacIsaac and Perkins were developed in patients with type 2 diabetes and, therefore, its performance should be more reliable in this group. However, these algorithms did not show better accuracy and precision than other equations not developed in type 2 diabetes (Table 2). Thus, the fact that a formula has been developed in a specific clinical condition seems not to have an impact on the reliability of estimated GFR.

The utility of estimated GFR is a matter of debate. Several studies have evaluated the reliability of formulas based on creatinine and/or cystatin-C in patients with diabetes [15]. Interestingly, formulas failed more frequently in subjects with renal disease (Supplementary Table S6), a population in which a reliable evaluation of GFR is particularly needed. Our study is in line with previous reports. Gaspari et al. evaluated a group of 15 creatinine-based formulas in 600 patients with type 2 diabetes and showed very low concordance with measured GFR (CCC < 0.52; TDI average ± 50%) [13]. Illadis et al. observed that 20%–80% of estimated GFR based on creatinine and cystatin-C showed an error >30% of real renal function [11]. Similarly, Rossing et al. observed wide limits of agreement between estimated GFR and measured GFR for renal function at baseline and changes over time in diabetic patients with microalbuminuria and proteinuria [5].

This study has strengths and limitations. To the best of our knowledge, this is the first study that evaluated all the available creatinine and/or cystatin-C based formulas in type 2 diabetes. In addition, we used modern and potent statistical tools such as the agreement method proposed by Lin et al. [19,20,21] to evaluate the reliability of these equations. Our population was entirely Caucasian and, therefore, we cannot extrapolate the results to other populations like African Americans or Asians populations.

## 5. Conclusions

Formulas failed in reflecting properly the renal function in patients with type 2 diabetes. Caution is needed with the use of these formulas in clinical practice in patients with diabetes, a population at high risk for kidney disease. Whenever possible, the use of a gold standard method to measure real renal function is recommended.

## Figures and Tables

**Table 1:** Clinical characteristics of the patients included in the study.

N	475
Age (years)	63.1 ± 11.2
Gender (male-%)	333 (70.1)
Clinical condition (n—%)	
With renal disease	290 (61.1)
Diabetic nephropathy	120 (25.3)
Kidney transplantation	112 (23.6)
Nephroangioesclerosis	33 (6.9)
Glomerulonephritis	11 (2.3)
Acquired Dominant Polycystic Kidney Disease	8 (1.7)
Intertitial nephritis	6 (1.3)
Without renal disease	185 (38.9)
Heart failure	34 (7.2)
Cirrhosis	33 (6.9)
Liver transplantation	12 (2.5)
Other	106 (22.3)
measured GFR mean ± SD (mL/min)	57.3 ± 36.3
measured GFR range (mL/min)	8.5–180.6
CKD stages (n—%)	
1 (>90 mL/min)	86 (18.1)
2 (60–90 mL/min)	94 (19.8)
3 (30–60 mL/min)	156 (32.8)
4 (15–30 mL/min)	116 (24.4)
5 (<15 mL/min)	23 (4.9)
Height (m)	1.68 ± 0.09
Weight (kg)	85.2 ± 18.4
Body Mass Index (kg/m^2^)	30.2 ± 5.6
Body Surface Area (m^2^)	1.94 ± 0.22
Serum Creatinine (mg/dL)	1.82 ± 1.15
Serum Cystatin-C (g/dL)	1.84 ± 0.96
24 h creatinine clearance median (IQR) (mL/min)	41.8 (40.4)
24-h proteinuria median (IQR) (mg/24 h)	427.7 (1440.1)

**Table 2:** Analysis of agreement between estimated glomerular filtration rate (GFR) using creatinine- and cystatin C–based formulas and measured GFR using plasma clearance of iohexol for the overall population of the study.

Formula	CCC	TDI	CP	Formula	CCC	TDI	CP
**Creatinine-based-formulas**							
Effersøe	0.91 (0.89)	61 (65)	25 (24)	aMDRD	0.92 (0.91)	59 (63)	27 (25)
Edward-White	0.85 (0.83)	82 (88)	20 (19)	Wright	0.88 (0.87)	70 (75)	22 (21)
Jelliffe-1	0.87 (0.85)	88 (95)	19 (18)	MCQ	0.88 (0.87)	77 (82)	22 (20)
Mawer	0.88 (0.87)	74 (79)	21 (20)	Sobh	0.85 (0.83)	87 (93)	18 (17)
Jelliffe-2	0.91 (0.90)	57 (61)	27 (26)	Virga	0.88 (0.87)	72 (77)	21 (20)
Cockcroft-Gault	0.89 (0.87)	70 (75)	22 (21)	CHUQ	0.84 (0.82)	96 (103)	18 (17)
Björnsson	0.87 (0.85)	80 (85)	19 (18)	CKD-EPI-cr	0.92 (0.91)	58 (62)	27 (25)
Mogensen	0.76 (0.73)	144 (156)	13 (12)	Lund-Malmö (LBM)	0.85 (0.83)	91 (97)	18 (17)
Hull	0.88 (0.87)	76 (81)	21 (20)	Lund-Malmö	0.91 (0.90)	59 (63)	26 (25)
Gates	0.91 (0.89)	66 (70)	24 (23)	Lund-1	0.91 (0.90)	53 (56)	29 (27)
Walser	0.90 (0.89)	64 (68)	25 (23)	Lund-2 (LBM)	0.81 (0.77)	107 (114)	14 (13)
Davis Chandler	0.89 (0.87)	68 (73)	24 (22)	Lund-Malmö (Rv)	0.92 (0.91)	55 (59)	28 (26)
Baracskay	0.82 (0.80)	79 (85)	21 (20)	Lund-Malmö (RvLBM)	0.88 (0.86)	74 (79)	21 (20)
Martin	0.83 (0.81)	96 (102)	14 (13)	FAS-cr	0.83 (0.81)	93 (98)	15 (13)
**Cystatin-C-based**							
Le Bricon	0.82 (0.80)	81 (87)	19 (17)	Jonsson	0.92 (0.91)	59 (64)	26 (25)
Tan	0.92 (0.90)	54 (58)	28 (27)	Stevens-1	0.93 (0.92)	49 (53)	31 (29)
Hoek	0.92 (0.91)	51 (55)	29 (28)	Stevens-2	0.93 (0.92)	47 (50)	32 (30)
Larsson	0.93 (0.92)	49 (53)	30 (29)	Tidman	0.91 (0.90)	61 (66)	26 (24)
Perkins	0.74 (0.71)	119 (126)	8 (7)	Grubb-2009	0.86 (0.84)	98 (107)	18 (17)
Orebro	0.86 (0.84)	96 (104)	18 (17)	Hojs	0.88 (0.86)	70 (74)	21 (20)
Grubb-2005	0.88 (0.87)	85 (92)	20 (19)	Grubb-2014 (CAPA)	0.93 (0.92)	52 (56)	29 (27)
Rule-cy	0.91 (0.90)	60 (65)	26 (24)	CKD-EPI-cy	0.93 (0.92)	51 (55)	30 (28)
MacIsaac	0.89 (0.88)	62 (66)	24 (23)	FAS-cy	0.82 (0.80)	86 (92)	17 (15)
Arnal-Dade	0.93 (0.92)	53 (57)	29 (27)	24 h-CrCl	0.81 (0.77)	85 (94)	20 (18)
**Creatinine-cystatin-C-based-formulas**							
Ma	0.93 (0.91)	54 (58)	27 (26)	CKD-EPI-cr-cy	0.94 (0.94)	45 (49)	32 (31)
Stevens	0.95 (0.94)	44 (47)	33 (32)	FAS-cr-cy	0.85 (0.83)	78 (83)	16 (14)

CCC: Corcondance correlation coefficient; TDI: Total Deviation Index; CP: Coverage probability. 24 h-CrCl: 24 h creatinine clearance Results expressed for unadjusted GFR values by body surface area (BSA) (mL/min).

**Table 3:** Estimated and measured glomerular filtration rate (GFR) in a representative group of 14 diabetic subjects.

		Creatinine				Cystatin-C			Creatinine and Cystatin-C		
Case	mGFR	CG	aMDRD	MCQ	CKD-EPI	Rule	MacIsaac	CKD-EPI	Ma	Stevens	CKD-EPI
1	17	13 *	11 *	11 *	10 *	13 *	20	13 *	13 *	12 *	11 *
2	17	26	24	22	23	23	35	25	28	25	23
3	27	22	16	15	15	25	37	27	24	21	20
4	28	34	31	33	33	30	43	35	38	34	33
5	48	57	56	63	55	41	57	44	59	52	48
6	49	36	29 *	27 *	28 *	47	65	53	44	38	38
7	67	41 *	47 *	50 *	44 *	43 *	59 *	45 *	54 *	48 *	44 *
8	68	142	82	110	91	47 *	65	57 *	79	72	70
9	99	80 *	65 *	81 *	68 *	108	127	127	103	88 *	94
10	97	131	133	95	105	78 *	95	80 *	123	109	94
11	116	154	159	131	124	117	132	125	172	152	129
12	118	95	106	120	100	96	113	108	125	109	106
13	150	299	206	182	171	130	151	159	209	188	167
14	151	201	115	133	133	137	154	141	153	134	139

CG: Cockroft-Gault. aMDRD: Abbreviated Modification of Diet in Renal Disease. BIS: Berlin Initiative Study. CKD-EPI: Chronic Kidney Disease Epidemiology Collaboration. MCQ: Mayo Clinic Quadratic. * Estimations of GFR that misclassified the patient as one CKD stage lower. Estimations of GFR that misclassified the patient as one CKD stage higher.

## Supplementary Materials

The following are available online at https://www.mdpi.com/2077-0383/8/10/1543/s1, Figure S1: Bland-Altman plots of the difference between the GFR values measured by the plasma clearance of iohexol (mGFR) and a representative number of 10 formulas based on creatinine and/or cystatin-C versus the mean of both; Supplementary Figure S2: Classification of the patients in CKD stages by a representative group of creatinine and/or cystatin-C based formulas; Supplementary Table S1: Analysis of the agreement between estimated GFR using creatinine- and cystatin C–based formulas and measured GFR using plasma clearance of iohexol for the overall population of the study; Supplementary Table S2: Analysis of agreement between estimated GFR using creatinine- and cystatin C–based formulas and measured GFR using plasma clearance of iohexol for four subgroups of diabetics based on their mGFR values (<30, 30–60, 60–90, >90 mL/min); Supplementary Table S3: Diverse degrees of overestimation or underestimation when comparing estimated GFR by aMDRD formula with measured GFR. Greyed out, hyperfiltering patients (mGFR > 120 mL/min) who were not detected by eGFR; Supplementary Table S4: (P10) Proportion of eGFR results within 10% of mGFR values. P(30) Proportion of eGFR results within 30% of mGFR values. (Unadjusted); Supplementary Table S5: (P10) Proportion of eGFR results within 10% of mGFR values, P(30) proportion of eGFR results within 30% of mGFR values. (Adjusted). Supplementary Table S6: Clinical characteristics of the patients included in the study in whom bias between estimated and measured GFR was lower or higher than 10%.

## References

[1] Zhang P., Gregg E. (2017). Global economic burden of diabetes and its implications. Lancet Diabetes Endocrinol..

[2] Schaubel D.E., Morrison H.I., Desmeules M., Parsons D.A., Fenton S.S. (1999). End-stage renal disease in Canada: Prevalence projections to 2005. CMAJ.

[3] de Boer I.H., Rue T.C., Hall Y.N., Heagerty P.J., Weiss N.S., Himmelfarb J. (2011). Temporal trends in the prevalence of diabetic kidney disease in the United States. JAMA.

[4] Perkins B.A., Nelson R.G., Ostrander B.E., Blouch K.L., Krolewski A.S., Myers B.D., Warram J.H. (2005). Detection of renal function decline in patients with diabetes and normal or elevated GFR by serial measurements of serum cystatin C concentration: Results of a 4-year follow-up study. J. Am. Soc. Nephrol..

[5] Rossing P., Rossing K., Gaede P., Pedersen O., Parving H.H. (2006). Monitoring kidney function in type 2 diabetic patients with incipient and overt diabetic nephropathy. Diabetes Care.

[6] Rigalleau V., Lasseur C., Perlemoine C., Barthe N., Raffaitin C., de La Faille R., Combe C., Gin H. (2006). A simplified Cockcroft-Gault formula to improve the prediction of the glomerular filtration rate in diabetic patients. Diabetes Metab..

[7] Fontseré N., Salinas I., Bonal J., Bayés B., Riba J., Torres F., Rios J., Sanmartí A., Romero R. (2006). Are prediction equations for glomerular filtration rate useful for the long-term monitoring of type 2 diabetic patients?. Nephrol. Dial. Transplant..

[8] Beauvieux M.C., Le Moigne F., Lasseur C., Raffaitin C., Perlemoine C., Barthe N., Chauveau P., Combe C., Gin H., Rigalleau V. (2007). New predictive equations improve monitoring of kidney function in patients with diabetes. Diabetes Care.

[9] Pucci L., Triscornia S., Lucchesi D., Fotino C., Pellegrini G., Pardini E., Miccoli R., Del Prato S., Penno G. (2007). Cystatin C and estimates of renal function: Searching for a better measure of kidney function in diabetic patients. Clin. Chem..

[10] Li H.X., Xu G.B., Wang X.J., Zhang X.C., Yang J.M. (2010). Diagnostic accuracy of various glomerular filtration rates estimating equations in patients with chronic kidney disease and diabetes. Chin. Med. J. (Engl.).

[11] Iliadis F., Didangelos T., Ntemka A., Makedou A., Moralidis E., Gotzamani-Psarakou A., Kouloukourgiotou T., Grekas D. (2011). Glomerular filtration rate estimation in patients with type 2 diabetes: Creatinine- or cystatin C-based equations?. Diabetologia.

[12] Inker L.A., Schmid C.H., Tighiouart H., Eckfeldt J.H., Feldman H.I., Greene T., Kusek J.W., Manzi J., Van Lente F., Zhang Y.L. (2012). CKD-EPI Investigators. Estimating glomerular filtration rate from serum creatinine and cystatin C. N. Engl. J. Med..

[13] Gaspari F., Ruggenenti P., Porrini E., Motterlini N., Cannata A., Carrara F., Jiménez Sosa A., Cella C., Ferrari S., Stucchi N. (2013). GFR Study Investigators. The GFR and GFR decline cannot be accurately estimated in type 2 diabetics. Kidney Int..

[14] MacIsaac R.J., Ekinci E.I., Premaratne E., Lu Z.X., Seah J.M., Li Y., Boston R., Ward G.M., Jerums G. (2015). The Chronic Kidney Disease-Epidemiology Collaboration (CKD-EPI) equation does not improve the underestimation of Glomerular Filtration Rate (GFR) in people with diabetes and preserved renal function. BMC Nephrol..

[15] Porrini E., Ruggenenti P., Luis-Lima S., Carrara F., Jiménez A., de Vries A.P.J., Torres A., Gaspari F., Remuzzi G. (2019). Estimated GFR: Time for a critical appraisal. Nat. Rev. Nephrol..

[16] Luis-Lima S., Marrero-Miranda D., González-Rinne A., Torres A., González-Posada J.M., Rodríguez A., Salido E., Aldea-Perona A., Gaspari F., Carrara F. (2015). Estimated Glomerular Filtration Rate in Renal Transplantation: The Nephrologist in the Mist. Transplantation.

[17] Luis-Lima S., Gaspari F., Negrín-Mena N., Carrara F., Díaz-Martín L., Jiménez-Sosa A., González-Rinne F., Torres A., Porrini E. (2018). Iohexol plasma clearance simplified by dried blood spot testing. Nephrol. Dial. Transpl..

[18] Du Bois D., Du Bois E.F. (1989). A formula to estimate the approximate surface area if height and weight be known. 1916. Nutrition.

[19] Lin L.I. (1989). A concordance correlation coefficient to evaluate reproducibility. Biometrics.

[20] Lin L., Hedayat A.S., Wu W. (2012). A Comparative Model for Continuous and Categorical Data. Statistical Tools for Measuring Agreement.

[21] Lin L., Hedayat A.S., Sinha B., Yang M. (2002). Statistical methods in assessing agreement. J. Am. Stat. Assoc..

[22] Altman D.G. (1991). Practical Statistics for Medical Research.

[23] National Kidney Foundation (2002). K/DOQI clinical practice guidelines for chronic kidney disease: Evaluation, classification, and stratification. Am. J. Kidney Dis..

[24] Perrone R.D., Madias N.E., Levey A.S. (1992). Serum creatinine as an index of renal function: New insights into old concepts. Clin. Chem..

[25] Jones J.D., Burnett P.C. (1974). Creatinine metabolism in humans with decreased renal function: Creatinine deficit. Clin. Chem..

[26] Tomlinson B.E., Walton J.N., Rebeiz J.J. (1969). The effects of ageing and of cachexia upon skeletal muscle. A histopathological study. J. Neurol. Sci..

[27] Bleiler R.E., Schedl H.P. (1962). Creatinine excretion: Variability and relationships to diet and body size. J. Lab. Clin. Med..

[28] Knight E.L., Verhave J.C., Spiegelman D., Hillege H.L., de Zeeuw D., Curhan G.C., de Jong P.E. (2004). Factors influencing serum cystatin-C levels other than renal function and the impact on renal function measurement. Kidney Int..

