# Supplementary material for: The Error of Estimated GFR in Type 2 Diabetes Mellitus
Source: J Clin Med. 2019 Sep 26;8(10):1543. doi: 10.3390/jcm8101543 (PMC6832380; doi:10.3390/jcm8101543)
Supplement: Supplementary file 1 [file jcm-08-01543-s001.pdf]

The error of estimated GFR in type 2 Diabetes Mellitus

Supplementary information

Supplementary Figure S1........ **Page 2**
Bland-Altman plots of the difference between the GFR values measured by the plasma clearance of iohexol (mGFR) and a representative number of 10 formulas based on creatinine and/or cystatin-C versus the mean of both.

Supplementary Figure S2........ **Page 3**
Classification of the patients in CKD stages by a representative group of creatinine and/or cystatin-C based formulas.

Supplementary Table S1 .... **Page 4**
Analysis of agreement between estimated GFR using creatinine- and cystatin C–based formulas and measured GFR using plasma clearance of iohexol for the overall population of the study.

Supplementary Table S2........ **Page 5**
Analysis of agreement between estimated GFR using creatinine- and cystatin C–based formulas and measured GFR using plasma clearance of iohexol for 4 subgroups of diabetics based on their mGFR values (<30, 30-60, 60-90, > 90 ml/min).

Supplementary Table S3........ **Page 6**
Diverse degrees of overestimation or underestimation when comparing estimated GFR by aMDRD formula with measured GFR. Greyed out, hyperfiltering patients (mGFR > 120 ml/min) who were not detected by eGFR.

Supplementary Table S4........ **Page 7**
(P10) proportion of eGFR results within 10% of mGFR values. P(30) proportion of eGFR results within 30% of mGFR values. (Unadjusted)

Supplementary Table S5........ **Page 8**
(P10) proportion of eGFR results within 10% of mGFR values, P(30) proportion of eGFR results within 30% of mGFR values. (Adjusted)

Supplementary Table S6........ **Page 9**
Clinical characteristics of the patients included in the study in whom bias between estimated and measured GFR was lower or higher than 10%.

Figure S1. Bland-Altman plots of the difference between the GFR values measured by the plasma clearance of iohexol (mGFR) and a representative number of 10 formulas based on creatinine and/or cystatin-C versus the mean of both.

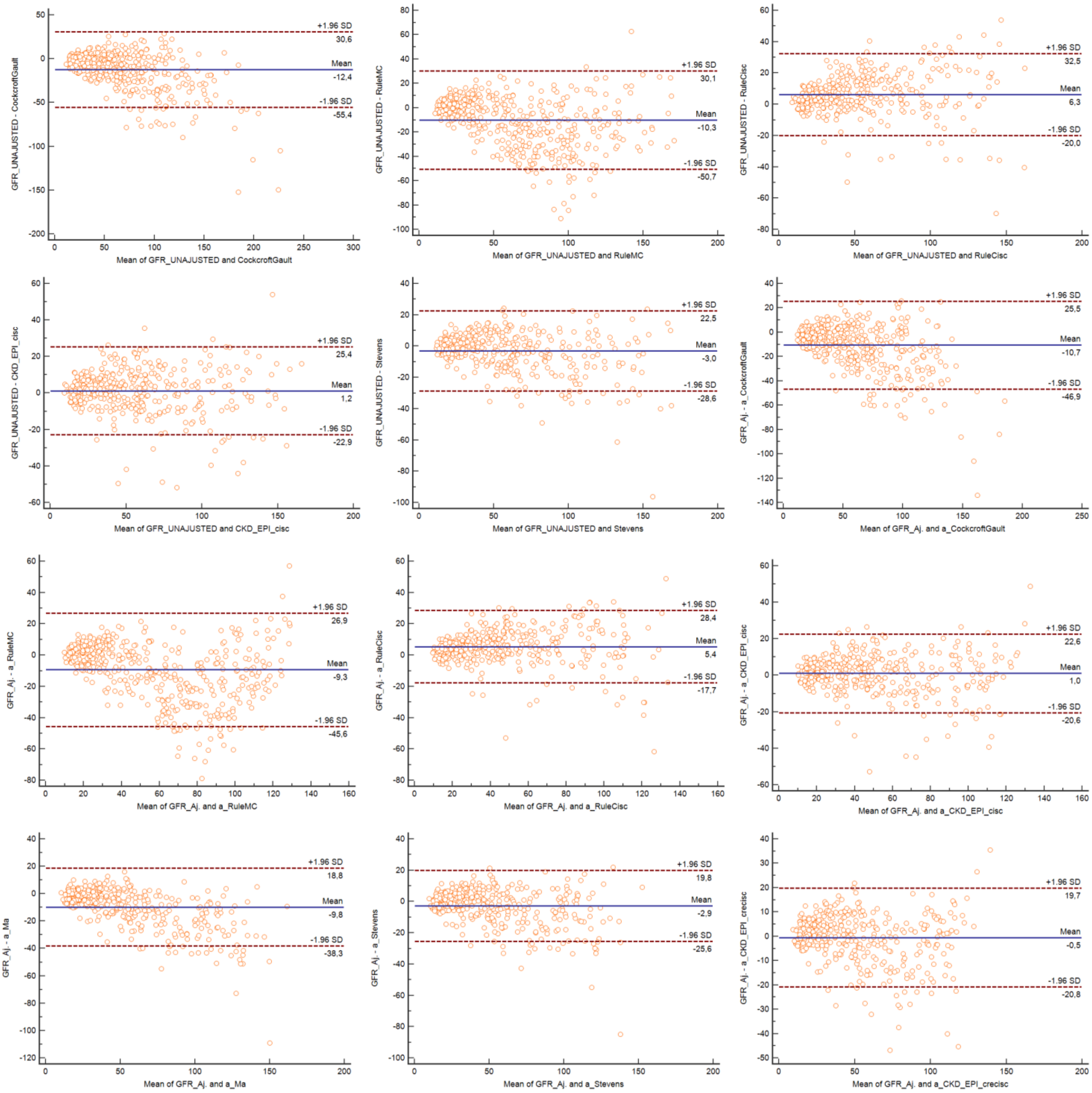

Bland-Altman plots of the difference between the GFR values measured by the plasma clearance of iohexol (mGFR) and a representative number of 10 formulas based on creatinine and/or cystatin-C versus the mean of both. The straight and the dashed lines indicate mean difference and 95% limits of agreement, respectively. Results expressed for unadjusted (ml/min) and adjusted GFR values by BSA (ml/min/1.73m^2)

Figure S2. Classification of the patients in CKD stages by a representative group of creatinine and/or cystatin-C based formulas.

Cockcroft-Gault

Stage	GFR	N	True positive	False positive	Missing
1	86	128	78 (91%)	50 (39%)	8 (9%)
2	94	90	41 (44%)	49 (54%)	53 (56%)
3	156	152	97 (62%)	55 (36%)	59 (38%)
4	116	100	69 (59%)	31 (31%)	47 (41%)
5	23	5	1 (4%)	4 (80%)	22 (96%)

aMDRD

Stage	GFR	N	True positive	False positive	Missing
1	86	113	78 (91%)	35 (31%)	8 (9%)
2	94	89	44 (47%)	45 (51%)	50 (53%)
3	156	131	90 (58%)	41 (31%)	66 (42%)
4	116	122	82 (71%)	40 (33%)	34 (29%)
5	23	20	8 (35%)	12 (60%)	15 (65%)

MCQ

Stage	GFR	N	True positive	False positive	Missing
1	86	156	83 (97%)	73 (47%)	3 (3%)
2	94	85	32 (34%)	53 (62%)	62 (66%)
3	156	87	58 (37%)	29 (33%)	98 (63%)
4	116	122	78 (67%)	44 (36%)	38 (33%)
5	23	25	7 (30%)	18 (72%)	16 (70%)

CKD-EPI_cr

Stage	GFR	N	True positive	False positive	Missing
1	86	122	80 (93%)	42 (34%)	6 (7%)
2	94	86	44 (47%)	42 (49%)	50 (53%)
3	156	121	88 (56%)	33 (27%)	68 (44%)
4	116	121	81 (70%)	40 (33%)	35 (30%)
5	23	25	9 (39%)	16 (64%)	14 (61%)

Perkins

Stage	GFR	N	True positive	False positive	Missing
1	86	121	71 (83%)	50 (41%)	15 (17%)
2	94	112	31 (33%)	81 (72%)	63 (67%)
3	156	140	46 (29%)	94 (67%)	110 (71%)
4	116	17	8 (7%)	9 (53%)	108 (93%)
5	23	0	0 (0%)	0 (-)	23 (100%)

MCQ_cy

Stage	GFR	N	True positive	False positive	Missing
1	86	58	55 (64%)	3 (5%)	31 (36%)
2	94	56	32 (34%)	24 (43%)	62 (66%)
3	156	139	96 (62%)	43 (31%)	60 (38%)
4	116	109	73 (63%)	36 (33%)	43 (37%)
5	23	28	12 (52%)	16 (57%)	11 (48%)

MacIsaac

Stage	GFR	N	True positive	False positive	Missing
1	86	88	69 (80%)	19 (22%)	17 (20%)
2	94	93	46 (49%)	47 (51%)	48 (51%)
3	156	136	83 (53%)	53 (39%)	73 (47%)
4	116	69	50 (43%)	19 (28%)	66 (57%)
5	23	4	4 (17%)	0 (0%)	19 (83%)

CKD-EPI_cy

Stage	GFR	N	True positive	False positive	Missing
1	86	75	66 (77%)	9 (12%)	20 (23%)
2	94	65	42 (45%)	23 (35%)	52 (55%)
3	156	123	92 (59%)	31 (25%)	64 (41%)
4	116	109	77 (66%)	32 (29%)	39 (34%)
5	23	18	9 (39%)	9 (50%)	14 (61%)

Ma

Stage	GFR	N	True positive	False positive	Missing
1	86	99	71 (83%)	28 (28%)	15 (17%)
2	94	82	42 (45%)	40 (49%)	52 (55%)
3	156	113	85 (54%)	28 (25%)	71 (46%)
4	116	90	72 (62%)	18 (20%)	44 (38%)
5	23	6	4 (17%)	2 (33%)	19 (83%)

Stevens

Stage	GFR	N	True positive	False positive	Missing
1	86	80	67 (78%)	13 (16%)	19 (22%)
2	94	80	46 (49%)	34 (43%)	48 (51%)
3	156	112	86 (55%)	26 (23%)	70 (45%)
4	116	101	75 (65%)	26 (26%)	41 (35%)
5	23	17	9 (39%)	8 (47%)	14 (61%)

CKD-EPI_crcy

Stage	GFR	N	True positive	False positive	Missing
1	86	81	69 (80%)	12 (15%)	17 (20%)
2	94	75	46 (49%)	29 (39%)	48 (51%)
3	156	103	83 (53%)	20 (19%)	73 (47%)
4	116	106	77 (66%)	29 (27%)	39 (34%)
5	23	25	12 (52%)	13 (52%)	11 (48%)

The error of estimated GFR in type 2 Diabetes Mellitus

Classification of the patients in CKD stages by a representative group of creatinine and/or cystatin-C-based formulas. *True positives cases* represent the subjects that were correctly classified in each CKD stages by eGFR; *False positives cases* represent the patients who were classified in one CKD stage based on eGFR when actually belong to a different stage; and *missing cases* represent the cases that were not classified in the corresponding CKD stage.

* The percentage of false positive cases refers to the number of cases defined in each CKD stages by measured GFR (grey column).

** The percentage of true positive and missing cases refers to the number of cases defined in each CKD stages by estimated GFR.

Table S1. Analysis of agreement between estimated GFR using creatinine- and cystatin C–based formulas and measured GFR using plasma clearance of iohexol for the overall population of the study

**Creatinine-based-formulas**							
	CCC	TDI	cp		CCC	TDI	cp
Effersøe	0.90 (0.89)	61 (65)	25 (24)	aMDRD	0.92 (0.90)	59 (63)	27 (25)
Edward-White	0.85 (0.83)	82 (88)	20 (19)	Wright	0.88 (0.86)	70 (75)	22 (21)
Jelliffe-1	0.86 (0.84)	88 (95)	19 (18)	MCQ	0.88 (0.87)	77 (82)	22 (20)
Mawer	0.88 (0.86)	74 (79)	21 (20)	Sobh	0.84 (0.82)	87 (93)	18 (17)
Jelliffe-2	0.91 (0.90)	57 (61)	27 (26)	Virga	0.88 (0.86)	72 (77)	21 (20)
Cockcroft-Gault	0.88 (0.87)	69 (74)	22 (21)	CHUQ	0.84 (0.82)	96 (103)	18 (17)
Björnsson	0.86 (0.84)	80 (85)	19 (18)	CKD-EPI-cr	0.92 (0.90)	58 (62)	27 (25)
Mogensen	0.76 (0.73)	145 (156)	13 (12)	Lund-Malmö (LBM)	0.84 (0.82)	91 (97)	18 (17)
Hull	0.88 (0.86)	75 (81)	21 (20)	Lund-Malmö	0.91 (0.90)	59 (63)	26 (25)
Gates	0.90 (0.89)	66 (70)	24 (23)	Lund-1	0.91 (0.90)	53 (56)	29 (27)
Walser	0.90 (0.88)	64 (68)	25 (23)	Lund-2 (LBM)	0.80 (0.77)	107 (114)	14 (13)
Davis Chandler	0.89 (0.87)	68 (73)	24 (22)	Lund-Malmö (Rv)	0.92 (0.91)	55 (59)	28 (26)
Baracskay	0.82 (0.80)	79 (85)	21 (20)	Lund-Malmö (RvLBM)	0.87 (0.86)	74 (79)	21 (20)
Martin	0.82 (0.80)	96 (102)	14 (13)	FAS-cr	0.82 (0.80)	93 (98)	15 (13)
**Cystatin-C-based**							
	CCC	TDI	CP		CCC	TDI	CP
Le Bricon	0.81 (0.79)	81 (87)	19 (17)	Jonsson	0.92 (0.91)	59 (64)	26 (25)
Tan	0.91 (0.90)	54 (58)	28 (27)	Stevens-1	0.93 (0.91)	49 (53)	31 (29)
Hoek	0.92 (0.90)	51 (55)	29 (28)	Stevens-2	0.93 (0.92)	47 (50)	32 (30)
Larsson	0.93 (0.92)	49 (53)	30 (29)	Tidman	0.91 (0.90)	61 (66)	26 (24)
Perkins	0.73 (0.71)	119 (126)	8 (7)	Grubb-2009	0.86 (0.84)	98 (106)	18 (17)
Orebro	0.85 (0.83)	96 (104)	18 (17)	Hojs	0.88 (0.86)	70 (74)	21 (20)
Grubb-2005	0.88 (0.87)	85 (92)	20 (19)	Grubb-2014 (CAPA)	0.93 (0.91)	52 (56)	29 (27)
Rule-cy	0.91 (0.89)	60 (64)	26 (24)	CKD-EPI-cy	0.93 (0.92)	51 (55)	30 (28)
MacIsaac	0.89 (0.87)	62 (66)	24 (23)	FAS-cy	0.81 (0.79)	86 (92)	17 (15)
Arnal-Dade	0.93 (0.91)	53 (57)	29 (27)				
**Creatinine-cystatin-C-based-formulas**							
	CCC	TDI	CP		CCC	TDI	CP
Ma	0.92 (0.91)	54 (58)	27 (26)	CKD-EPI-cr-cy	0.94 (0.93)	45 (49)	32 (31)
Stevens	0.95 (0.94)	44 (47)	33 (32)	FAS-cr-cy	0.85 (0.83)	78 (83)	16 (14)

CCC: Corcondance correlationcoefficient; TDI: Total Deviation Index; CP: Coverage probability. Results expressed for adjusted GFR values by BSA (ml/min/1.73m^2)

Table S2. Analysis of agreement between estimated GFR using creatinine- and cystatin C–based formulas and measured GFR using plasma clearance of iohexol for 4 subgroups of diabetics based on their mGFR values (<30, 30-60, 60-90, > 90 ml/min).

Equation	< 30 ml/min (n=139)			30-60 ml/min (n=155)			60-90 ml/min (n=95)			> 90 ml/min (n=86)		
**Creatinine (Cr)**	**CCC**	**TDI**	**CP**	**CCC**	**TDI**	**CP**	**CCC**	**TDI**	**CP**	**CCC**	**TDI**	**CP**
CG	0.41 (0.31)	82 (93)	19 (17)	0.46 (0.38)	70 (78)	22 (20)	0.25 (0.16)	64 (75)	24 (21)	0.53 (0.43)	53 (62)	27 (23)
aMDRD	0.48 (0.37)	68 (77)	23 (21)	0.55 (0.48)	60 (68)	25 (23)	0.31 (0.22)	59 (68)	26 (23)	0.60 (0.48)	37 (43)	37 (32)
MCQ	0.45 (0.33)	78 (89)	21 (19)	0.42 (0.36)	98 (112)	18 (16)	0.21 (0.13)	71 (82)	21 (18)	0.55 (0.42)	35 (41)	39 (35)
CKD-EPI	0.47 (0.36)	70 (80)	22 (20)	0.53 (0.46)	67 (76)	23 (21)	0.34 (0.24)	48 (56)	30 (27)	0.71 (0.61)	24 (28)	52 (46)
**Cystatin-C (Cy)**	**CCC**	**TDI**	**CP**	**CCC**	**TDI**	**CP**	**CCC**	**TDI**	**CP**	**CCC**	**TDI**	**CP**
Rule	0.48 (0.37)	72 (83)	22 (20)	0.48 (0.39)	60 (68)	24 (22)	0.29 (0.20)	57 (66)	23 (19)	0.48 (0.33)	40 (48)	34 (30)
MacIsaac	0.35 (0.26)	101 (115)	15 (13)	0.49 (0.40)	52 (59)	27 (24)	0.51 (0.39)	30 (35)	45 (39)	0.56 (0.42)	32 (38)	42 (37)
CKD-EPI	0.51 (0.40)	68 (79)	23 (21)	0.56 (0.47)	51 (58)	29 (26)	0.42 (0.30)	39 (46)	35 (31)	0.67 (0.55)	26 (31)	48 (43)
**Cr and Cy**	**CCC**	**TDI**	**CP**	**CCC**	**TDI**	**CP**	**CCC**	**TDI**	**CP**	**CCC**	**TDI**	**CP**
Ma	0.54 (0.43)	62 (71)	24 (22)	0.56 (0.48)	54 (61)	27 (24)	0.43 (0.33)	42 (49)	32 (28)	0.41 (0.30)	50 (58)	22 (17)
Stevens	0.59 (0.49)	53(61)	28 (25)	0.63(0.55)	46 (52)	31 (28)	0.53 (0.43)	33 (39)	40 (35)	0.62 (0.49)	31 (36)	42 (37)
CKD-EPI	0.57 (0.47)	58 (67)	26 (23)	0.61 (0.54)	49 (56)	30 (27)	0.53 (0.43)	32 (38)	41 (36)	0.73 (0.63)	22 (26)	55 (48)

CCC: Corcondance correlation coefficient; TDI: Total Deviation Index; CP: Coverage probability. Results expressed for unadjusted GFR values by BSA (ml/min)

Table S3. Diverse degrees of overestimation or underestimation when comparing estimated GFR by aMDRD formula with measured GFR. Greyed out, hyperfiltering patients (mGFR > 120 ml/min) who were not detected by eGFR.

Case	mGFR	CG	aMDRD	MCQ	CKD-EPI-cr	RuleCisc	MacIsaac	CKD_EPI-cy	Ma	Stevens	CKD-EPI-crcy
1	120	102	133	105	100	137	145	116	168	145	115
2	121	111	117	133	113	102	119	120	137	121	118
3	122	155	132	142	124	156	167	146	180	157	142
4	122	141	121	147	121						
5	122	134	115	107	113	84	102	92	120	106	103
6	123	145	119	112	117	98	116	108	132	116	114
7	124	151	111	150	123	92	111	112	127	113	118
8	124	96	101	124	102						
9	128	129	109	140	114	163	178	153	164	140	139
10	129	137	131	133	117	123	139	129	159	139	127
11	129	106	97	95	100	105	116	103	124	109	105
12	129	176	161	138	129	123	139	131	177	157	135
13	130	170	126	166	136	108	128	135	148	132	136
14	130	182	135	163	136	97	118	116	144	128	126
15	131	160	138	107	119	94	110	106	140	125	115
16	131	121	130	119	108	132	143	123	163	142	122
17	132	168	166	136	128	132	148	134	185	163	136
18	135	163	134	146	128						
19	136	150	161	131	124	114	130	123	171	151	127
20	137	166	142	112	125	133	145	126	170	150	133
21	141	182	154	161	142	97	117	118	156	139	130
22	141	220	161	170	150	120	140	147	177	158	150
23	142	257	150	152	159	182	195	170	206	181	172
24	144	163	140	151	131	133	150	143	171	150	141
25	147	142	157	130	122						
26	147	166	127	165	136	130	149	150	162	142	146
27	148	166	163	136	127	117	134	128	173	153	131
28	148	173	129	164	136	131	148	149	164	145	146
29	150	300	206	182	172	130	151	159	209	188	167
30	151	201	115	133	133	137	154	141	153	134	139
31	157	217	153	184	156	113	135	141	168	150	149
32	163	221	238	136	147						
33	165	180	127	169	139	126	146	151	160	141	147
34	173	236	185	111	132	120	134	120	184	163	134
35	174	167	139	164	139	151	167	158	182	159	153
36	174	279	270	148	163						
37	181	188	157	156	139						

Results expressed for unadjusted GFR values by BSA (ml/min).

Table S4. (P10) proportion of eGFR results within 10% of mGFR values. P(30) proportion of eGFR results within 30% of mGFR values.

Creatinine based formulas					
**Formula**	**P10**	**P30**	**Formula**	**P10**	**P30**
Effersøe	30.7	69.7	aMDRD	27.4	72.6
Edward-White	25.5	58.9	Wright	28.0	65.1
Jelliffe-1	21.3	55.4	MCQ	19.6	59.6
Mawer	22.9	58.9	Sobh	24.8	56.0
Jelliffe-2	28.2	72.4	Virga	24.8	61.1
Cockcroft-Gault	24.8	63.2	CHUQ	17.1	53.5
Björnsson	23.8	56.2	CKD-EPI-cr	31.4	71.6
Mogensen	14.1	40.0	Lund-Malmö (LBM)	18.1	53.3
Hull	22.9	59.2	Lund-Malmö	25.3	72.0
Gates	26.3	68.0	Lund-1	29.7	77.5
Walser	30.3	67.2	Lund-2 (LBM)	16.6	45.3
Davis Chandler	24.4	67.2	Lund-Malmö (Rv)	29.3	74.7
Baracskay	20.4	62.1	Lund-Malmö (RvLBM)	23.8	60.6
Martin	16.0	44.6	FAS-cr	15.2	48.8
**Cystatin-C based formulas**					
**Formula**	**P10**	**P30**	**Formula**	**P10**	**P30**
Le Bricon	24.1	57.9	Jonsson	30.0	78.7
Tan	32.1	78.2	Stevens-1	37.4	82.8
Hoek	36.7	81.5	Stevens-2	40.5	83.8
Larsson	37.7	82.6	Tidman	30.3	71.0
Perkins	7.2	27.9	Grubb-2009	21.5	51.5
Orebro	15.9	51.5	Hojs	22.8	61.0
Grubb-2005	23.6	62.3	Grubb-2014 (CAPA)	33.6	82.6
Rule-cy	27.9	78.2	CKD-EPI-cy	37.4	81.5
MacIsaac	31.5	70.0	FAS-cy	21.0	50.8
Arnal-Dade	33.1	82.3			
**Creatinine Cystatin-C based formulas**					
**Formula**	**P10**	**P30**	**Formula**	**P10**	**P30**
Ma	32.3	71.3	CKD-EPI-cr-cy	38.2	84.9
Stevens	36.4	85.4	FAS-cr-cy	18.5	51.3

Results expressed for unadjusted GFR values by BSA (ml/min)

Table S5. (P10) proportion of eGFR results within 10% of mGFR values, P(30) proportion of eGFR results within 30% of mGFR values.

Creatinine based formulas					
**Formula**	**P10**	**P30**	**Formula**	**P10**	**P30**
Effersøe	30.7	69.7	aMDRD	27.4	72.6
Edward-White	25.5	58.9	Wright	28.0	65.1
Jelliffe-1	21.3	55.4	MCQ	19.6	59.6
Mawer	22.9	58.9	Sobh	24.8	56.0
Jelliffe-2	28.2	72.4	Virga	24.8	61.1
Cockcroft-Gault	24.8	63.2	CHUQ	17.1	53.5
Björnsson	23.8	56.2	CKD-EPI-cr	31.4	71.6
Mogensen	14.1	40.0	Lund-Malmö (LBM)	18.1	53.3
Hull	22.9	59.2	Lund-Malmö	25.3	72.0
Gates	26.3	68.0	Lund-1	29.7	77.5
Walser	30.3	67.2	Lund-2 (LBM)	16.6	45.3
Davis Chandler	24.4	67.2	Lund-Malmö (Rv)	29.3	74.7
Baracskay	20.4	62.1	Lund-Malmö (RvLBM)	23.8	60.6
Martin	16.0	44.6	FAS-cr	15.2	48.8
**Cystatin-C-based-formulas**					
**Formula**	**P10**	**P30**	**Formula**	**P10**	**P30**
Le Bricon	24.1	57.9	Jonsson	30.0	78.7
Tan	32.1	78.2	Stevens-1	37.4	82.8
Hoek	36.7	81.5	Stevens-2	40.5	83.8
Larsson	37.7	82.6	Tidman	30.3	71.0
Perkins	7.2	27.9	Grubb-2009	21.5	51.5
Orebro	15.9	51.5	Hojs	22.8	61.0
Grubb-2005	23.6	62.3	Grubb-2014 (CAPA)	33.6	82.6
Rule-cy	27.9	78.2	CKD-EPI-cy	37.4	81.5
MacIsaac	31.5	70.0	FAS-cy	21.0	50.8
Arnal-Dade	33.1	82.3			
**Creatinine-cystatin-C-based-formulas**					
**Formula**	**P10**	**P30**	**Formula**	**P10**	**P30**
Ma	32.3	71.3	CKD-EPI-cr-cy	38.2	18.5
Stevens	36.4	85.4	FAS-cr-cy	18.5	51.3

Results expressed for adjusted GFR values by BSA (ml/min/1.73m^2)

Table S6. Clinical characteristics of the patients included in the study in whom bias between estimated and measured GFR was lower or higher than 10%.

	**Bias lower than ±10%**	**Bias higher than**	p
N	149	326	
Age (y)	62.0 ± 10.6	63.6 ± 11.4	0.15
Gender (male-%)	110 (74.0)	223 (68.0)	0.14
**With renal disease**	70 (47.0)	220 (67.5)	<0.0001
**Without renal disease**	79 (53)	106 (32.5)	<0.0001
measured GFR mean ± SD	73.5 ± 38.7	50.2 ± 33.0	<0.0001
measured GFR range (ml/min)	12.1-173.8	8.5-180.6	<0.0001
Height (m)	1.69 ± 0.08	1.67 ± 0.09	<0.028
Weight (kg)	86.6 ± 17.3	84.7 ± 18.9	0.26
Body Mass Index (kg/m^2)	30.2 ± 5.4	30.1 ± 5.7	0.81
Body Surface Area (m^2)	1.97 ± 0.21	1.93 ± 0.23	0.10
Serum Creatinine (mg/dL)	1.46 ± 0.91	1.98 ± 1.2	<0.0001
Serum Cystatin-C (g/dL)	1.52 ± 0.80	1.98 ± 0.99	<0.0001
24 hour creatinine clearance	48.5 (35.4)	39.2 (40.8)	0.6
